# *Lernaea cyprinacea* Linnaeus, 1758 (Copepoda: Lernaeidae) infection on *Betta rubra* Perugia, 1893 (Anabantiformes: Osphronemidae) from Aceh Province, Indonesia

**DOI:** 10.1590/S1984-29612022015

**Published:** 2022-03-16

**Authors:** Firman Muhammad Nur, Agung Setia Batubara, Nur Fadli, Syamsul Rizal, Mohd Nor Siti-Azizah, Martin Wilkes, Zainal Abidin Muchlisin

**Affiliations:** 1 Graduate School of Mathematics and Applied Sciences, Universitas Syiah Kuala, Banda Aceh, Indonesia; 2 Faculty of Mathematics and Natural Sciences, Universitas Negeri Medan, Medan, North Sumatera, Indonesia; 3 Faculty of Marine and Fisheries, Universitas Syiah Kuala, Banda Aceh, Indonesia; 4 Institute of Marine Biotechnology, Universiti Malaysia Terengganu, Kuala Terengganu, Malaysia; 5 Center for Agroecology, Water and Resilience, Coventry University, Coventry, United Kingdom; 6 Department of Aquaculture, Faculty of Marine and Fisheries, Universitas Syiah Kuala, Banda Aceh, Indonesia

**Keywords:** Cot Bira, endangered species, endemic species, ornamental fish, parasite, population, Cot Bira, espécies ameaçadas, espécies endêmicas, peixes ornamentais, parasita, população

## Abstract

*Betta rubra* is an ornamental freshwater fish endemic to northern Sumatra, Indonesia. The *B. rubra* population has decreased in recent decades, and is classified as an endangered species in the IUCN Red List. This study aims to report for the first time infection by *L. cyprinacea* in *B. rubra* harvested from the Aceh Besar region of Indonesia. The fish samples were obtained from the Cot Bira tributaries, Aceh Besar District, Indonesia from January to December 2020. The results showed that the parasite infected 6 out of 499 samples in August and September, with a prevalence and intensity rate of 1% and 2 parasites/fish, respectively. The eyes and pectoral fins were the common infection sites. Despite *B. rubra* is not an optimal host (small size) for the parasite, this parasite might serve as additional threatening factors for the endangered *B. rubra* fish population.

## Resumo

*Betta rubra* é um peixe de água doce ornamental endemico da região norte Sumatra, Indonesia. A população de *Betta rubra* diminuiu ao longo dos anos, sendo classificada como espécie em extinção na Lista Vermelha da IUCN. Este estudo tem como objetivo relatar pela primeira vez infecção por *L. cyprinacea* em *B. rubra* coletados na região de Aceh Besar na Indonésia. As amostras de peixes foram obtidas nos afluentes Cot Bira, distrito de Aceh Besar, Indonésia de janeiro a dezembro de 2020. Os resultados mostraram que o parasito infectou 6 das 499 amostras em agosto e setembro, com uma prevalência e taxa de intensidade de 1% e 2 parasitas/peixes, respectivamente. Os olhos e as nadadeiras peitorais foram os sítios de infecção mais comuns. Apesar de *B. rubra* não ser um hospedeiro ideal (pequeno tamanho) para o parasita, este parasita pode servir como fator de ameaça adicional para a população de peixes *B. rubra*, ameaçada de extinção.

## Introduction

*Betta rubra* is a fish species within the family Osphronemidae. Etymologically, the “rubra” is Latin referring to “ruber” which means red, describing the bright red color pattern on the fish’s body. Furthermore, *B. rubra* is a popular ornamental freshwater fish, endemic to northern Sumatra, comprising Aceh to Sibolga (Fahmi et al., 2020; Nur et al., 2020; Nur et al., 2022b), which has been classified as an endangered fish species in the IUCN Red List (Low, 2019). The fish’s wild population is decreasing and becoming rare to find in nature, due to habitat perturbation (Muchlisin, 2008), low recruitment (Batubara et al., 2020), and high parasite loadings. Generally, parasites are present in the environment and the body of fishes, hence can cause infection when there is an imbalance in the host-parasite relationship (Martins et al., 2002). Parasite development is influenced by several factors, including climate change (Aleuy & Kutz, 2020), temperature increase (Cereja et al., 2018; Hossain et al., 2013; Piasecki et al., 2004), organic enrichment (Muchlisin et al., 2014; Welicky et al., 2017; Yen Le et al., 2014), acidification and dissolved oxygen (Dalu et al., 2012; Sánchez-Hernández, 2017). Parasites can become problematic in fish hosts with compromised immunity specially in artificial environments (e.g. fish farms), affecting fish health, growth, reproduction, and survival (Lieke et al., 2020; Opiyo et al., 2018; Segar et al., 2018).

*Lernaea cyprinacea* Linnaeus, 1758, known as anchor worm, is a common parasite of freshwater and marine fishes. It is a cosmopolitan ectoparasite belonging to the copepod group, and does not have a specific host (McAllister et al., 2011; Piasecki et al., 2004). Generally, *L. cyprinacea* embed its anchor into the hosts’ body to suck blood. In fish, the parasites usually penetrate the skin, fins, and eyes. This parasite was first reported from the Eurasian region, and it spread globally through fish introduced into various countries (Hassan et al., 2008; Tidd, 1934; Walter & Boxshall, 2021).

Infections by *L. cyprinacea* can cause pathogenesis and mortality due to bleeding from the infected organs (Carnevia & Speranza, 2003; Silva-Souza et al., 2000), leading to secondary infections caused by bacteria and fungi (Abbas et al., 2014; Boxshall & Defaye, 2008; Fast, 2014). Although the parasite is not common in temperate areas (Ahnelt et al., 2018; Bednarska et al., 2009; Piasecki et al., 2004), recent reports suggest that its distribution now includes temperate lakes in Europe (Ahnelt et al., 2018). The expansion of its geographic distribution may be related to climate change and intensity of introduced species (Muchlisin, 2008; Waicheim et al., 2019).

*Lernaea cyprinacea* has infected several species of freshwater and marine fish in various countries, including South Africa (Chakona et al., 2019; Welicky et al., 2017), Argentina (Plaul et al., 2010; Salinas et al., 2016), Asia (Innal & Avenant-Oldewage, 2012), Spain (Sánchez-Hernández, 2017), Siberia (Schäperclaus et al., 1991), Europe (Ahnelt et al., 2018; Stavrescu-Bedivan et al., 2011), Jepang (Nagasawa et al., 2007), Brazil (Narciso et al., 2019; Santos et al., 2020) and Israel (Lahav & Sarig, 1964). Infection occurs in capture and ornamental fisheries, and affects both farmed and wild populations (Mancini et al., 2008). This ectoparasite infects fishes of various sizes and ages (Barson et al., 2008; Gutiérrez-Galindo & Lacasa-Millán, 2005). In Indonesia, infection has been reported in goldfish *Carassius auratus* (Kriswijayanti, 2014), Arowana *Scleropages jardinii* (Shatrie et al., 2011), Gourami *Osphronemus gourami* (Kismiyati & Wulan Sari, 2014), Koi *Cyprinus rubrofuscus* (Wardany & Kurniawan, 2014), Gobi *Sicyopus zosterophorum* (Adriany et al., 2020), *Lentipes mekonggaensis* (Adriany et al., 2020), common carp *Cyprinus carpio* (Sarimudin et al., 2016; Winaruddin & Eliawardani, 2007), Tilapia *Oreochromis niloticus* (Ulkhaq et al., 2018), and catfish (Fautama, 2018; Salsabilla, 2021; Ulkhaq et al., 2018). Generally, these fish are infested in aquaculture systems. However, there has been no report of the infection in wild fish, especially ornamental fish in Aceh. Therefore, this study aims to report for the first time infection by *L. cyprinacea* in *B. rubra* harvested from Aceh, Indonesia.

## Materials and Methods

### Host sampling

**Figure 1:**
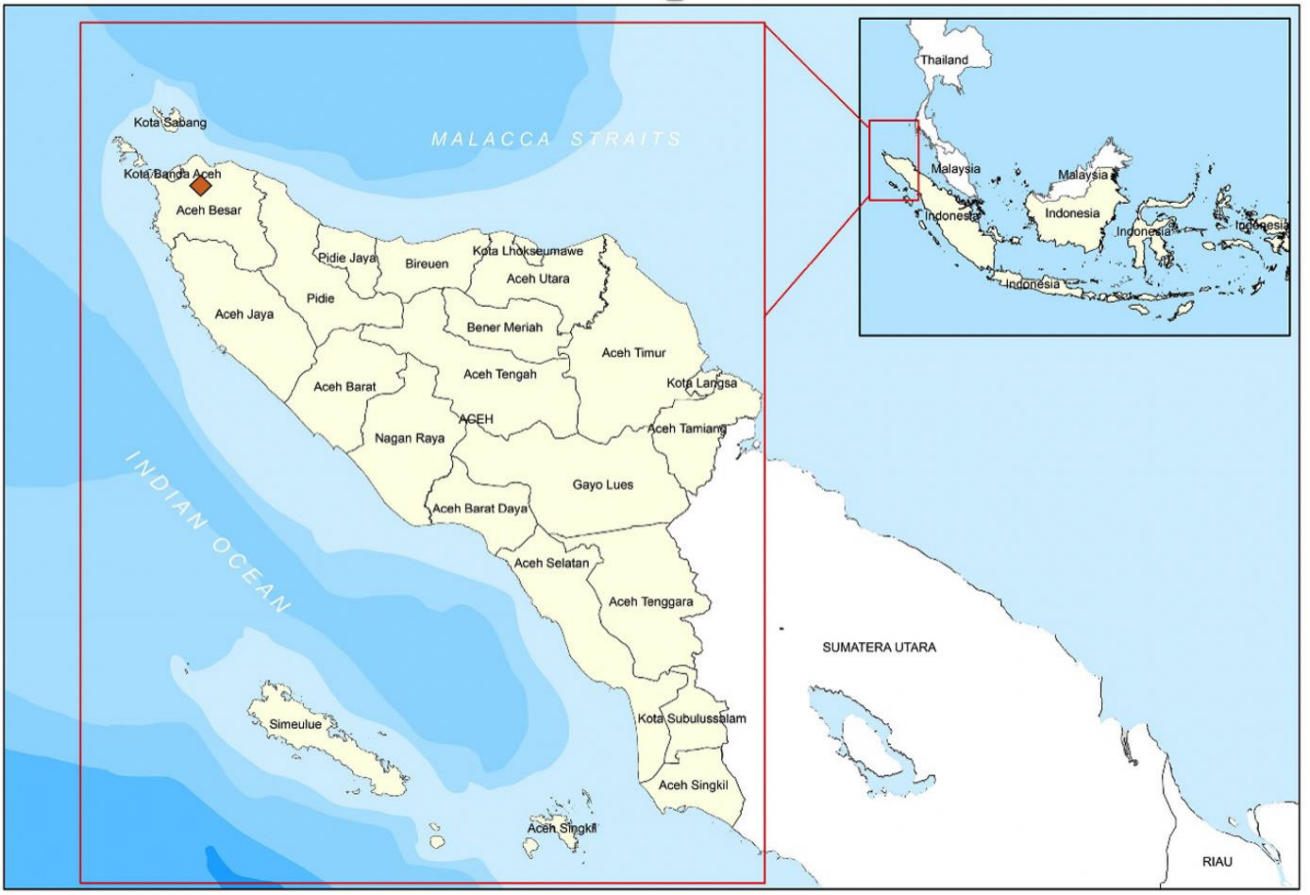
The map of Aceh province showed the sampling location (highlighted in red box). Indonesia map insert.

The survey was conducted in Cot Bira tributaries (GPS coordinate, 05° 29.895' and 095° 27.939') Aceh Besar District, Aceh province, Indonesia, (Figure 1), from January to December 2020. Hand-nets were used to obtain the fish samples at one-week intervals for 12 months, and sampling was done from 08.00 AM to 6.00 PM. The samples were kept in live condition in a 100 mL plastic bag filled with water and oxygen. Each bag was then stocked with 1 fish to avoid cross-infection, then kept in an icebox at 24-26 °C. The samples were transported to the Laboratory of Ichthyology, Faculty of Marine and Fisheries, Syiah Kuala University, Banda Aceh for further analysis. Taxonomic identification was based on Kottelat et al. (1993). Samples were euthanized by immersing the fish in cold water (4 °C) for 5 min, then preserved in 10% formalin (Nur et al., 2022a). These procedures were conducted in compliance with Research Ethics Guideline of Universitas Syiah Kuala No. 958/2015.

### Parasite observation procedure and data analysis

A total of 499 fish samples were collected, and measured for total length (mm) and body weight (g). Subsequently, the external body of the samples was examined, including the head section, left and right sides of the body, fins, operculum, and nose using a Stereo Microscope (Euromax Stereoblue, Type SB. 1902, Made in the Netherlands). Tweezers were used to collect the parasites, which were preserved in 70% ethanol. The parasites were identified based on Avenant-Oldewage & Robinson (1996), and photographed for documentation. Prevalence and intensity of infection were calculated according to Bush et al. (1997).

## Results

### Prevalence and intensity

**Figure 2:**
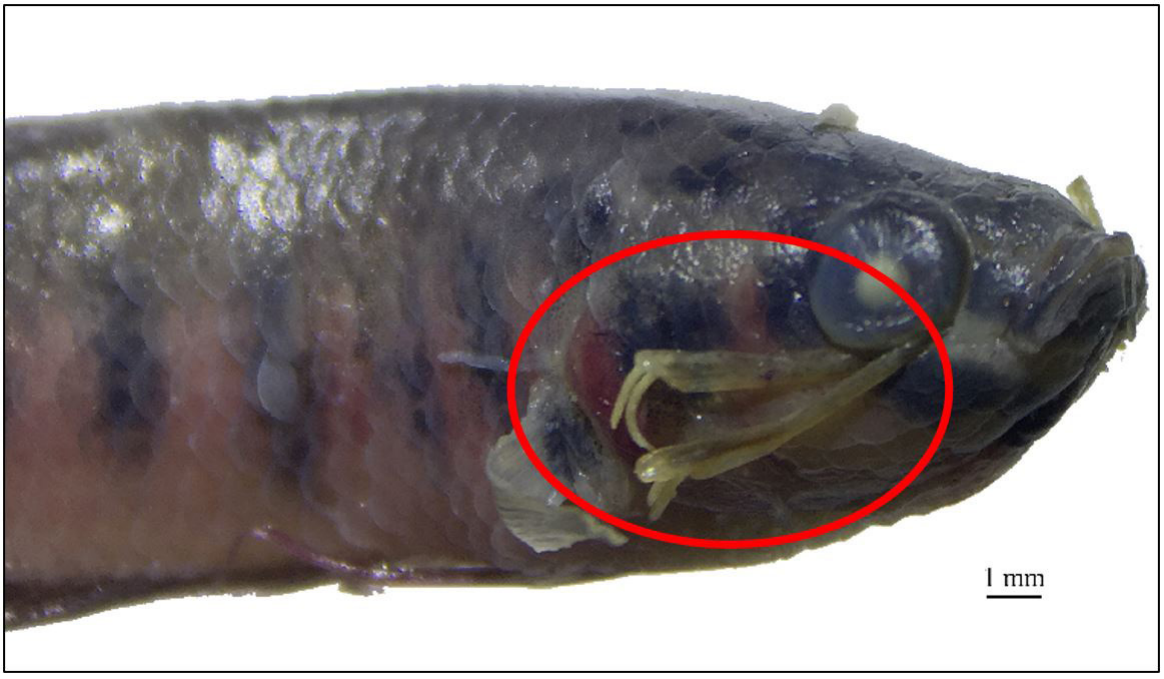
*Betta rubra* specimen infected by the anchor worm, *Lernaea cyprinacea*. The parasite is highlighted in the red circle.

**Figure 3:**
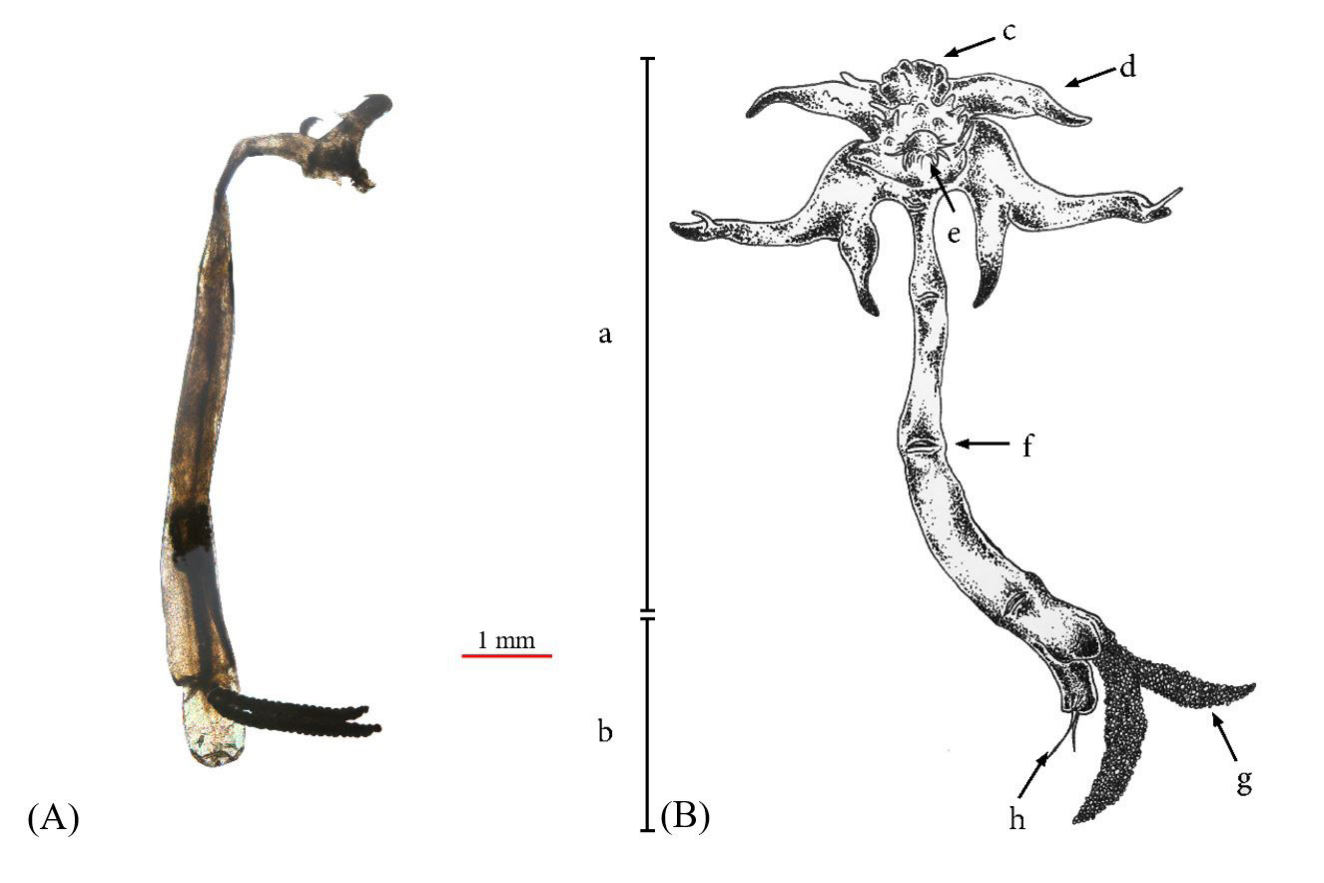
(A) The female anchor worm *Lernaea cyprinacea*. (B) The illustration of the female *L. cyprinacea* describes the body part of the: (a) anterior part, (b) posterior part, (c) antenna, (d) anchor, (e) the ventral anchor, (f) maxillary, (g) egg yolk sac, and (h) uropod.

A total of 6 samples (1%) exhibited *L. cyprinacea* infection. The infected fish were discovered in August and September, with the highest prevalence of 10% occurring in August. Table 1 showed that the average intensity was 2 parasites/fish. Figure 2 illustrates that the parasite infected the eyes, skin, pectoral, dorsal, and ventral fins. The highest prevalence was observed in four organs, namely the eyes (2 out of 499), pectoral (2 out of 499), dorsal (2 out of 499), ventral fins (2 out of 499) and skin (1 out of 499), while the highest intensity was found in the eyes and pectoral fins (1.5). The ectoparasites found in *B. rubra* were identified as *L. cyprinacea* (Figure 3) based on the presence of the following characteristics, according to Avenant-Oldewage & Robinson (1996): transparent to brownish-yellow body color, length ranging from 9.79 to 10.93 mm, antennas (c), maxillary, and an anterior holdfast (a), which consists of two pairs of anchors with an average length of 1.23±0.07 mm (d), posterior end provided with egg sacs and uropods (b)

**Table 1:** Prevalence and intensity infection of the anchor worm *Lernaea cyprinacea* according to sampling time.

**Parameter**	**Jan**	**Feb**	**Mar**	**Apr**	**May**	**June**	**July**	**Aug**	**Sept**	**Oct**	**Nov**	**Dec**	**Total**
Total sample (N)	22	25	50	50	50	47	37	50	50	50	50	18	499
Infected sample (Ni)	0	0	0	0	0	0	0	5	1	0	0	0	6
Total parasite (n)	-	-	-	-	-	-	-	8	2	-	-	-	10
Prevalence (%)	-	-	-	-	-	-	-	8.0	2.1	-	-	-	1.0
Intensity (Tot.parasite/fish)	-	-	-	-	-	-	-	2.0	2.0	-	-	-	2.0

### Habitat description

The tributary of the Bira Cot has a depth ranging from 14 to 45 cm, and a river width of 0.5 to 1.5 m with a slow current. The in situ measurements of the water quality parameters showed that the temperature, pH, and dissolved oxygen ranged from 24.3- 28.8 °C, 7.22 - 8.56, and 7.8-8.8 ppm, respectively (Table 2).

**Table 2:** Water temperature, pH and dissolved oxygen at the Cot Bira Tributaries.

**Parameter**	**Month**												**Range**
	**Jan**	**Feb**	**Mar**	**Apr**	**May**	**June**	**July**	**Aug**	**Sep**	**Oct**	**Nov**	**Dec**	
pH	8.27±0.1	8.17±0.1	8.18±0.1	7.77±0.2	7.64±0.2	7.81±0.1	7.80	7.51±0.3	7.65±0.4	7.43±0.1	8.16±0.2	8.37±0.1	7.22-8.56
Temperature (°C)	26±0.8	0.4	27.6±1.4	26.2±0.4	25.9±0.5	26.7±0.3	25.2±0.9	26.2±0.7	27.1±0.6	27.5±0.7	28.1±1.0	28.0±0.6	24.3-28.8
Dissolved oxygen (mg L^-1^)	8.2±0.1	8.1±0.2	8.6±0.2	8.6±0.2	8.5±0.1	8.2±0.2	8.4±01	8.3±0.1	8.1±0.2	8.2±0.2	7.9±0.1	8.2±0.1	7.8-8.8

## Discussion

This study showed that the prevalence and intensity of *L. cyprinacea* on *Betta rubra* was low, and the infection was observed in August and September. This is probably due to water temperature variation, which was at 26-27 °C during times when peak infection rates occurred, slightly higher than other months. According to Piasecki et al. (2004), the optimal temperature for *L. cyprinacea* development ranges from 25 to 28 °C (Hossain et al., 2018; Plaul et al., 2010). According to Kupferberg et al. (2009), water temperatures above 20 °C increase *L. cyprinacea* infection. On the other hand, it has been demonstrated that lower temperature can decrease the prevalence and intensity of infections by this parasite (Plaul et al., 2010).

In the present study, the eyes and pectoral fins of *B. rubra* were the sites most infected by *L. cyprinacea*, followed by the skin, dorsal, and ventral fins. Several studies showed that this parasite commonly infects the gills, mouth, and nostrils (Abbas et al., 2014; Acosta et al., 2013; Gutiérrez-Galindo & Lacasa-Millán, 2005). Infections by *L. cyprinacea* in the cornea or tissues around the eye of fishes can lead to fibrosis and bleeding (Eagle, 2012), as well as vision impairment and blindness (Padrós et al., 2018; Shariff, 1985; Uzmann & Rayner, 1958), making the host more susceptible to predation (Ubels et al., 2018). The fact that the eyes are a common infection site could be due to the cornea having many blood vessels and a thin membrane, being easier for the parasite to penetrate.

*Lernaea cyprinacea* can damage the scales of fishes by penetrating the skin into the muscles (Bozorgnia et al., 2018), hence infection of fish larvae will cause imminent death (Innal et al., 2017). Infected fish typically become malnourished following infection (Salinas et al., 2019; Sayyadzadeh & Joladeh-Roudbar, 2014; Smit et al., 2017). Furthermore, parasites deeply attached and embedded in the fish's body are difficult to release (Furtado et al., 2019). According to Mirzaei (2015), *L. cyprinacea* infection forms a wound that is easily infected by opportunistic bacteria, such as *Aeromonas hydrophila* (Hossain et al., 2013; Mancini et al., 2006). Also, infection of the gills causes epithelial proliferation and increases the spread of bacterial infection (Faruk, 2018; Gjessing et al., 2019; Shariff et al., 1986).

The observed prevalence and intensity of *L. cyprinacea* infection in *B. rubra* were relatively low in this study. This is in line with a study on *B. splendens* in Brazil, where the occurrence was due to the small size of the benthic fish (Santos et al., 2020). Ahnelt et al. (2018) obtained similar results in *Knipowitschia panizzae* and *Pomatoschistus canestrinii*, where the small benthic fishes had a short life cycle of one year and low infection rate. Nagasawa et al. (2007) also reported low infection rates in the barbell steed (*Hemibarbus labeo)*, dark chub (*Zacco temminckii)*, and the small Amur catfish (*Silurus asotus)*. This was related to the short life cycle and small body surface area of these species, which limits the attachment of the parasite (Hua et al., 2019; Raibaut et al., 1998). In the present study, the size of the fish host *B. rubra* ranged from 28.82–37.17 mm.

According to Tokşen et al. (2014), *L. cyprinacea* is commonly found in aquatic habitats with low water velocity and warm water temperatures, as observed in this study. The field observations showed that *B. rubra* habitat in Aceh Besar was damaged due to industrialization and channel engineering, causing topographic changes and silting of tributaries. This results in reduced depth and flow, increasing the temperature and possibly facilitating this ecto-parasite’s infection routes and reproduction. Therefore, it is necessary to address degradation in the tributaries and restore the *B. rubra* habitat to reduce the chances of infections by *L. cyprinacea*.

## Conclusion

This study is the first record of *L. cyprinacea* in a natural population of *B. rubra* from Indonesia. The parasitic prevalence was categorized as occasional (1%) with a low intensity level of 2 parasites/fish, occurring in August and September. The eyes and pectoral fins were the most commonly infected sites on hosts.
